# The impact of the COVID-19 pandemic on pediatric operations: a retrospective study of Chinese children

**DOI:** 10.1186/s13052-020-00915-3

**Published:** 2020-10-16

**Authors:** Yi Wei, Chengjun Yu, Tian Xin Zhao, Tao Lin, H. E. Dawei, Sheng-de Wu, Guang-hui Wei

**Affiliations:** 1 Department of Urology, Children’s Hospital of Chongqing Medical University, Room 806, Kejiao Building (NO.6 Building), No.136, 2nd Zhongshan Road, Yuzhong District, Chongqing City, 40014 China; 2 Chongqing Key Laboratory of Children Urogenital Development and Tissue Engineering, Chongqing City, China; 3 Ministry of Education Key Laboratory of Child Development and Disorders, Room 806, Kejiao Building (NO.6 Building), No.136, 2nd Zhongshan Roadm, Yuzhong District, Chongqing City, 400014 China; 4 China International Science and Technology Cooperation base of Child development and Critical Disorders, Chongqing City, China; 5 National Clinical Research Center for Child Health and Disorders, Chongqing City, China; 6 Chongqing Key Laboratory of Pediatrics Chongqing, Chongqing City, China

**Keywords:** COVID-19, Operation, Nucleic acid test, Children, China

## Abstract

**Background:**

The aim of this study was to quantify the impact of coronavirus disease 2019 (COVID-19) on pediatric operations, and establish preoperative, intraoperative, and postoperative protocols to improve the pediatric operations.

**Methods:**

We here compare the number of patients who underwent surgery in Chongqing Medical University Affiliated Children’s Hospital during the pandemic (January 23–March 11), after the pandemic (March 12–April 30), after our measures were put in place (May 1–May 21), and the equivalent period in 2019.

**Result:**

During the COVID-19 pandemic, 62.68% fewer patients underwent surgery than during the homologous period of time 1 year earlier (*P* < 0.01). After the COVID-19 pandemic, the number of orchidopexy cases increased significantly from 175.14 to 504.57 per week (*P* < 0.01). The large number of patients that accrued in our hospital may have increased the risk of COVID-19 transmission. In response, hospitals and clinics have made protocols and reorganized healthcare facilities (e.g., performing nucleic acid tests (NAT), adding adequate personal protective equipment (PPE)) from May 1, 2020. After the measures were implemented, the number of operations performed remained stable and comparable to the pre-pandemic period. COVID-19 RNA detection was performed in 5104 cases and there were no new confirmed cases in our hospital.

**Conclusion:**

This outbreak of COVID-19 has affected not only individuals with COVID-19 but also patients seeking surgical operations. Understanding the present situation helps clinicians provide a high level of treatment to all children.

## Introduction

COVID-19 is a novel coronavirus disease caused by the severe acute respiratory syndrome coronavirus 2 (SARS-CoV-2). The COVID-19 outbreak has spread globally and was declared a pandemic by the World Health Organization on March 11, 2020 [1]. As of June 11, more than 216 countries had reported cases of this illness, with a total of about 7,255,960 diagnosed cases causing over 412,583 deaths (World Health Organization, 2020).

China has enacted a series of strict measures since January 23, 2020 to block the quick spread of virus infection and control the severe epidemic [2]. This outbreak has affected not only individuals with COVID-19, but also patients seeking care for other conditions. A decrease in the number of patients seeking urological operations has been observed, indicating that COVID-19 significantly influenced people’s urological operation-seeking behavior [3, 4]. However, the influence of COVID-19 on the number of operations in other departments (emergency surgery, outpatient surgery, oncology surgery, gastrointestinal surgery, plastic surgery, hepatobiliary surgery, orthopedics, the cardio-thoracic department, and neurosurgery) in Chongqing China has yet to be explored. Whether new recommendations and measures could meet people’s needs for surgical services is also unknown.

Thus, assessing the present situation of pediatric surgery is helpful to the prediction of future surgical needs. It will also help surgical departments continue to provide adequate levels of treatment to patients with other conditions.

## Methods

This is an observational, retrospective, single-center study. Data were collected from patients who underwent surgery in Chongqing Medical University Affiliated Children’s Hospital from January 23, 2020 to May 21, 2020 and from the same period the previous year (from January 23, 2019 to May 21, 2019). The CDC of China and Chongqing Center for Disease Control and Prevention have reported that there were no new confirmed cases and no new suspected cases in Chongqing from February 25 to March 11 for over 2 weeks. Thus, we here define the COVID-19 pandemic period from January 23 to March 11 and the post-COVID-19 pandemic period from March 12 to May 21 in this study.

The number of children who underwent surgery in our surgical diagnosis and treatment center was determined. We made protocols and reorganized healthcare facilities to reduce the risk of transmission of COVID-19 in hospitals according to the state council regulation. We implemented those measures starting on May 1 (Table 1).

**Table 1:** Measures we had taken to reduce the risk of COVID-19 transmission in hospitals while keeping the recovered number of operations each week

	Our measures after the pandemic period	Our measures during the pandemic period
	Recovering the number of surgery	Reducing the number of surgery (The main measures we took)
1	Everyone should quarantine themselves at home for at least 14 days before coming to our hospital according to the guidelines offered by CDC^a^	No
2	Performing a risk assessment for each patient undergoing surgery	No (The risk evaluation system is not well formed during the pandemic)
3	Performing routine preoperative testing (e.g nucleic acid detection) among children and their parents and symptom screening is recommended to identify those with COVID-19^a^	No (Lack of enough Nucleic acid detection kit during the pandemic)
4	Providing enough PPE (e.g. gloves, mask, and eye protection.) for surgical operation team to protect the children, patients and healthcare workers^a^	Few (Lack of enough equipment during the pandemic)
5	Adding ultra low particulate air filters and anaesthesia air filter during the anaesthesia and operation	Yes
6	Performing thorough post-operative cleaning and sterilization with adequate time allowing the operating room air to be cycled after procedures (close the laminar flow system and use the medical air disinfection machine to ensure sterilization purification. Then restart the laminar flow system and ventilation equipment 2 h later.)	Yes
7.	Online medical services was recommended to reduce face-to-face contact^a^	No

*CDC* Centers for Disease Control and Prevention, *PPE* Personal protective equipment; ^a^: “new” from what we did before

Statistical analyses: The data were evaluated using IBM SPSS Statistics version 23 through descriptive and analytical statistics. We used ANOVA to examine the change in the number of cases presenting across the 17-week period. A *P*-value of less than 0.05 was considered statistically significant.

## Results

### Number of operations during the COVID-19 pandemic

During the COVID-19 pandemic, 62.86% fewer patients visited our hospital and underwent surgery relative to the corresponding period 1 year earlier (1226 vs 3301). We observed a statistically significant difference in the number of operations performed in different wards per week.

**Fig. 1:**
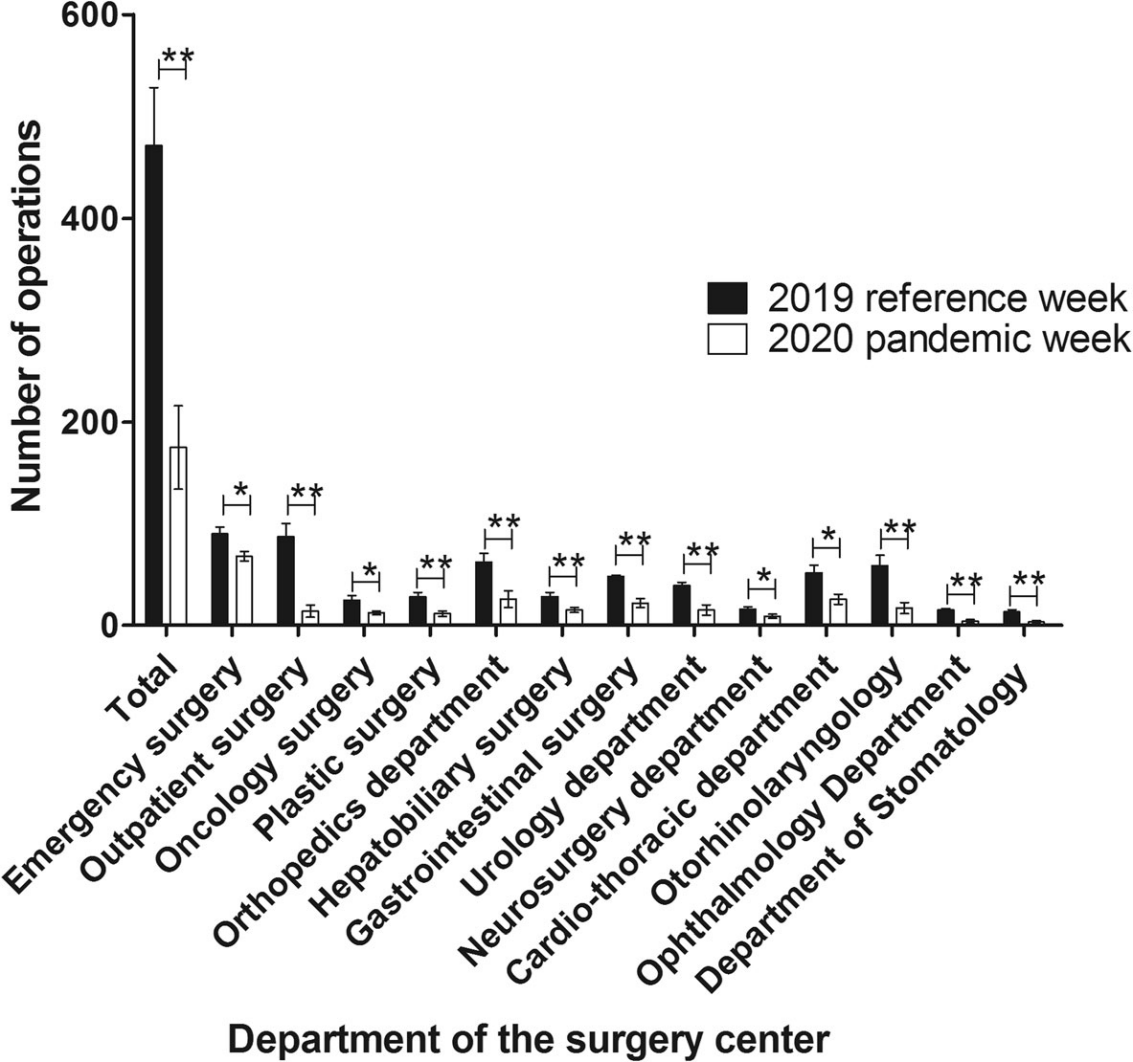
Children who underwent surgery during the COVID-19 pandemic compared to the corresponding period 1 year earlier. The number of operations performed by the emergency surgery, outpatient surgery, oncology surgery, plastic surgery, orthopedics department, hepatobiliary surgery, gastrointestinal surgery, urology department, neurosurgery department, cardio-thoracic department, otorhinolaryngology, ophthalmology department, and department of stomatology was significantly smaller during the pandemic period than during the corresponding period 1 year earlier. *P < 0.05, **P < 0.01

The number of emergency cases was also strongly affected by COVID-19; it decreased from 90.14 to 67.86 per week (*P* < 0.05) (Fig. 1).

### Number of operations after the COVID-19 pandemic

Based on these results, we speculated that people’s need for surgical services might have grown explosively in the post-COVID-19 period. Since Chongqing CDC reported that there were no new confirmed cases and no new suspected cases for over 2 weeks in Chongqing at March 11, children with congenital heart disease, pectus excavatum, diaphragmatic hernia, scoliosis, developmental dislocation of the hip, hypospadias, and cryptorchidism have come to our hospital and undergone surgery.

**Fig. 2:**
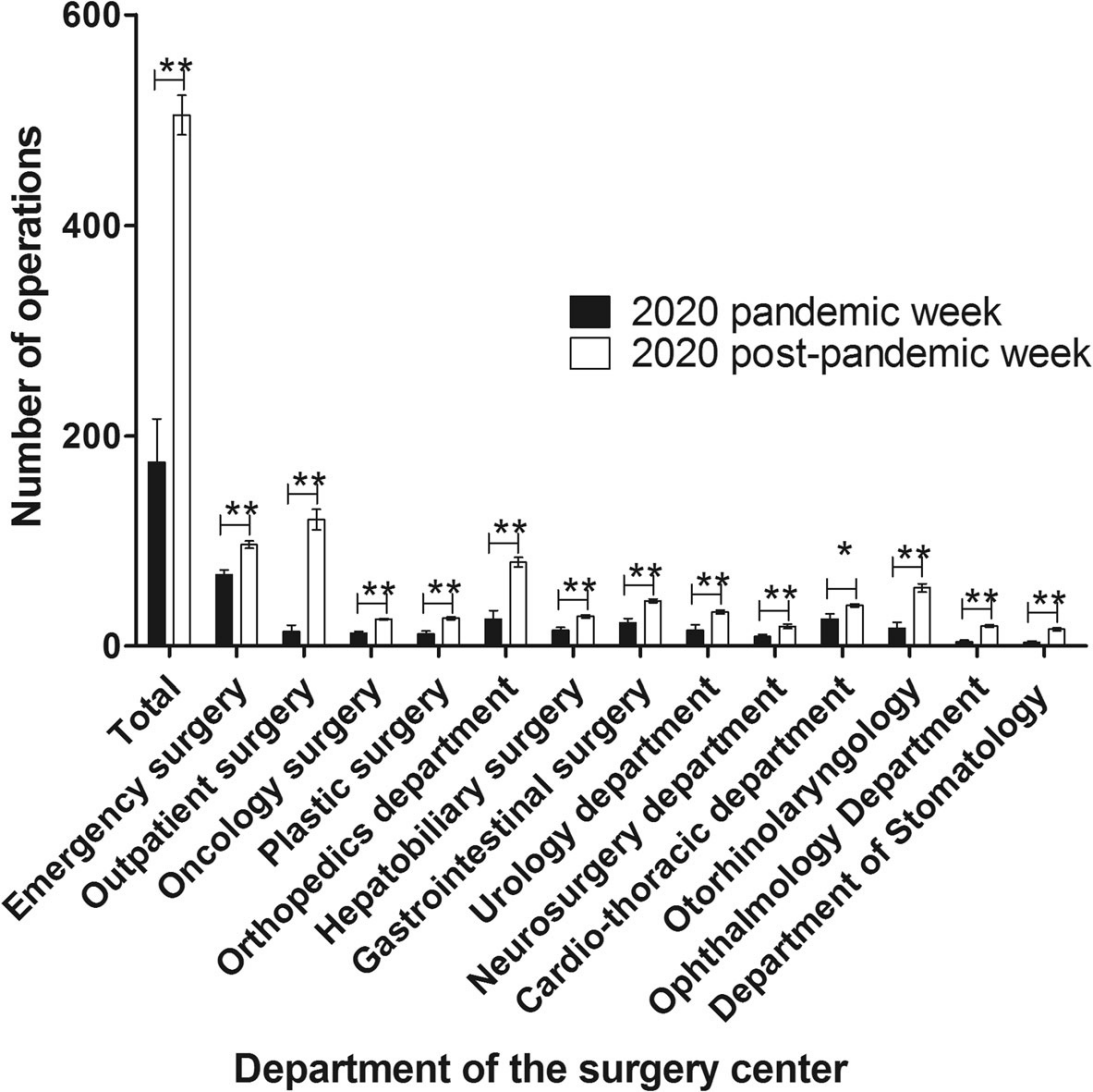
Numbers of operations performed during the pandemic and after the pandemic in 2020. There were significantly more operations performed by emergency operation, outpatient surgery, oncology surgery, plastic surgery, orthopedics department, hepatobiliary surgery, gastrointestinal surgery, urology department, neurosurgery department, cardio-thoracic department, otorhinolaryngology, ophthalmology department and department of stomatology during the post-pandemic than during the pandemic period. *P < 0.05, **P < 0.01

In our study, the number of operations increased significantly from 175.14 cases to 504.57 cases per week (*P* < 0.01). The number of emergency surgery also increased from 67.85 to 96.57 per week (*P* < 0.01) (Fig. 2).

### Recommendations for response to the COVID-19 crisis

However, the large number of patients who came to our hospital might have increased the risk of virus transmission. From a surgical perspective, we should find ways to reduce the risk of COVID-19 transmission in hospitals while keeping the recovered number of operations each week. Hence, our hospitals’ anesthesia department and operating room implemented measures based on patients, parents, and surgeons starting May 1, 2020 (Table 1).

### Number of operations and results of COVID-19 nucleic acid detection after implementation of anti-COVID-19 protocols

After implementation of anti-COVID-19 measures, the number of operations performed was recorded, and found to be 409, 474, and 416 each week, from May 1 to May 21 in 2020 and 434, 503, and 396 in the corresponding weeks of the previous year (*P*-value = 0.777). The COVID-19 nucleic acid test was negative in 100% (5104/5104) of the children who underwent surgery and in their parents.

## Discussion

As expected, there was an 62.86% decrease in the number of pediatric operations at our hospital during the COVID-19 pandemic relative the corresponding period 1 year earlier. The number of emergency surgical procedures was also strongly affected by COVID-19 and decreased from 90.14 to 67.86 per week. Our data strongly support the conclusion that COVID-19 significantly influenced people’s surgical care-seeking behavior. These facts were also corroborated by studies performed in other countries [3, 5]. Although this was not analyzed further, there are three likely possible reasons. First, the decreased number of surgeries might be attributed to the strict measures conducted by China. The strict measures include staying home and avoiding unnecessary outdoor activity and only traveling for work and to secure necessary health care. Reduced outdoor activity and travel may contribute to the decrease in the incidence of surgical emergencies. Second, children’s parents were apprehensive about viral transmission and chose to postpone seeking surgery. Third, during this period, we canceled such elective surgeries as cleft lip, pectus excavatum, hypospadias, and cryptorchidism during the pandemic to decrease the risk of COVID-19 transmission in our hospital.

After Chongqing CDC reported that, as of April 11, there had been no new confirmed cases and no new suspected cases for over 2 weeks in Chongqing, we observed a significant increase in the number of operations from 175.14 cases to 504.57 cases per week.

As of April 30, there are currently over 3,096,626 confirmed cases worldwide with total deaths exceeding 72,955 (https://covid19.who.int/). This pandemic is unlike anything that has been seen in recent history and is still uncontrolled worldwide. From a surgical perspective, many questions have arisen regarding the potential risks of the increasing operations. We should find ways to reduce the risk of transmission of COVID-19 in hospitals while continuing to meet the public’s need for surgery each week.

Some studies have recommended adopting a triage strategy to prevent wastage of medical resources and implement sufficient protection policies to prevent infection when treating COVID-19 patients [6, 7]. We made protocols and reorganized healthcare facilities according to state council regulations. After implementation, the number of operations performed by emergency surgery, outpatient surgery, oncology surgery, gastrointestinal surgery, plastic surgery, hepatobiliary surgery, orthopedic, cardio-thoracic, urology, and neurosurgery departments remained stable during the post-pandemic period. Furthermore, 5104 cases of COVID-19 RNA detection were performed and there were no new confirmed cases, no new suspected cases, and no existing confirmed cases in our hospital. Indicating that our measures may contribute to ensure the medical safety during the worldwide COVID pandemic.

The authors recognize that the present study has several limitations. First, it is a retrospective study with the associated inherent limitations. Second, this study was conducted with careful haste, so the analysis period was short and may not be representative. However, it provides a true representation of the effect that the COVID-19 pandemic has on pediatric surgery. We also described our experience-based protocols and reorganized healthcare facilities in response to the pandemic to improve the safety of surgery in our hospital, which is one of the largest specialist pediatric surgery centers in China. Our findings indicating that further measures should be taken to alleviate the health consequences caused by COVID-19. Recommendations include preoperative measures, intraoperative measures, and postoperative measures.

## Conclusion

The decreased number of pediatric operations at our hospital during the COVID-19 pandemic indicating that this outbreak has affected not only individuals with COVID-19, but also patients seeking care for other conditions. Recommendations and measures adapted to the local environment (e.g. quarantining at home for at least 14 days before coming to our hospital for surgery, performing risk-benefit assessment for every patient undergoing surgery, providing enough nucleic acid detection kits and personal protective equipment for all personnel and patients, and providing online medical services) is necessary to continue providing a high level of care to all children.

## Data Availability

Data sharing applicable to this article. And I wish to share my data.

## Abbreviations

COVID-19: Coronavirus disease 2019;
SARS-CoV-2: Severe acute respiratory syndrome coronavirus 2;
CDC: Center for disease control and prevention;
PPE: Personal protective equipment;
NAT: Nucleic acid test;
VSD: Ventricular septal defect;
DDH: Developmental dislocation of the hip;
UDT: Undescended testis
